# Signature RNAS and related regulatory roles in type 1 diabetes mellitus based on competing endogenous RNA regulatory network analysis

**DOI:** 10.1186/s12920-021-00931-0

**Published:** 2021-05-18

**Authors:** Qinghong Shi, Hanxin Yao

**Affiliations:** 1 Department of Clinical Laboratory, The Third Hospital of Jilin University, No. 126, Xiantai Street, Changchun, 130033 Jilin China; 2 Department of Clinical Laboratory, The First Hospital of Jilin University, No. 1, Xinmin Street, Chaoyang District, Changchun, 130021 Jilin China

**Keywords:** T1DM, LncRNAs, CeRNAs

## Abstract

**Background:**

Our study aimed to investigate signature RNAs and their potential roles in type 1 diabetes mellitus (T1DM) using a competing endogenous RNA regulatory network analysis.

**Methods:**

Expression profiles of GSE55100, deposited from peripheral blood mononuclear cells of 12 T1DM patients and 10 normal controls, were downloaded from the Gene Expression Omnibus to uncover differentially expressed long non-coding RNAs (lncRNAs), mRNAs, and microRNAs (miRNAs). The ceRNA regulatory network was constructed, then functional and pathway enrichment analysis was conducted. AT1DM-related ceRNA regulatory network was established based on the Human microRNA Disease Database to carry out pathway enrichment analysis. Meanwhile, the T1DM-related pathways were retrieved from the Comparative Toxicogenomics Database (CTD).

**Results:**

In total, 847 mRNAs, 41 lncRNAs, and 38 miRNAs were significantly differentially expressed. The ceRNA regulatory network consisted of 12 lncRNAs, 10 miRNAs, and 24 mRNAs. Two miRNAs (*hsa-miR-181a* and *hsa-miR-1275*) were screened as T1DM-related miRNAs to build the T1DM-related ceRNA regulatory network, in which genes were considerably enriched in seven pathways. Moreover, three overlapping pathways, including the phosphatidylinositol signaling system (involving *PIP4K2A*, *INPP4A*, *PIP4K2C*, and *CALM1*); dopaminergic synapse (involving *CALM1* and *PPP2R5C*); and the insulin signaling pathway (involving *CBLB* and *CALM1*) were revealed by comparing with T1DM-related pathways in the CTD, which involved four lncRNAs (*LINC01278*, *TRG-AS1*, *MIAT*, and *GAS5-AS1*).

**Conclusion:**

The identified signature RNAs may serve as important regulators in the pathogenesis of T1DM.

**Supplementary Information:**

The online version contains supplementary material available at 10.1186/s12920-021-00931-0.

## Background

Recently, type 1 diabetes mellitus (T1DM) is a multifactorial autoimmune disease characterized by insulin deficiency and hyperglycaemia, which is considered to involve the selective attack of insulin-producing pancreatic β cells by activated T lymphocytes that recognize their autoantigens [1]. T1DM accounts for approximately 5–10% of all cases of diabetes mellitus, and the incidence of T1DM is rising worldwide, with more than 80% of diabetes occurring in younger children [2, 3].

Many efforts have been made recently to gain insights into the pathogenesis of T1DM. Three main regions on chromosomes, including the protein tyrosine-phosphatase non-receptor-type 22 region on chromosome 1p13, the human leukocyte antigen region on chromosome 6p21, and the insulin region on chromosome 11p15, play essential roles in insulin expression, immune response, and β-cell function, which are associated with T1DM [4, 5]. More than 50 genomic risk loci have been identified for T1DM based on genome-wide association studies [5]. However, most of the risk loci are located in non-coding genomic regions, and an increasing number of studies have focused on the potential roles of long non-coding RNAs (lncRNAs) in pancreatic islets and the pathogenesis of T1DM [6, 7]. Motterle et al. found that some lncRNAs are modulated by proinflammatory cytokines during the development of T1DM in non-obese diabetic mice, which probably contributes to the sensitization of β cells to apoptosis and failure during the initial phases of T1DM [7]. The nuclear-enriched β-cell lncRNA *PLUTO* may regulate the expression of *PDX1*, which is a key pancreatic β-cell transcription factor; furthermore, knockdown of *PLUTO* is associated with the downregulation of *PDX1* in EndoC-βH1 cells and primary islet cells, implicating the roles of lncRNAs in the regulation of β-cell-specific transcription factors [8]. Therefore, examining the ability of lncRNAs to regulate gene expression and cell-specific transcription factors opens avenues to a better understanding of T1DM pathogenesis.

On the other hand, an increasing body of evidence indicates that microRNAs (miRNAs) play important roles in processes involved in the pathogenesis of T1DM, including immune system functions and β-cell metabolism and death [9, 10]. Assmann et al. have suggested that 11 circulating miRNAs (*miR-21-5p*, *miR-24-3p*, *miR-100-5p*, *miR-146a-5p*, *miR-148a-3p*, *miR-150-5p*, *miR-181a-5p*, *miR-210-5p*, *miR-342-3p*, *miR-375*, and *miR-1275*) are consistently dysregulated in T1DM patients [11]. It has been further revealed that five miRNAs (*miR-103a-3p*, *miR-155-5p*, *miR-200a-3p*, *miR-146a-5p*, and *miR-210-3p*), which have been confirmed as dysregulated miRNAs based on plasma miRNA expression profiles of T1DM patients and control individuals, could regulate genes involved in the innate immune system-, MAPK-, apoptosis-, insulin-, and cancer-related pathways [12]. Additionally, it is widely acknowledged that competing endogenous RNAs (ceRNAs) can interact with mRNAs by competing with miRNAs, and miRNA-mediated interactions between lncRNAs and mRNAs occur in the progression of various diseases [13–15]. However, few current studies have reported on the ceRNA-based regulatory mechanisms of T1DM. In the study of Yang et al. [16] global miRNA and mRNA expressions were profiled in peripheral blood mononuclear cells from 12 patients with newly diagnosed T1DM and 10 normal controls, while miRNA-mediated interactions between lncRNAs and mRNAs were not revealed. Thus, further studies aimed at clarifying ceRNA-based transcriptional signatures are needed to provide new insights into the pathogenesis of T1DM. In our present study, differentially expressed lncRNAs, mRNAs, and miRNAs between T1DM and normal controls were identified based on profiles retrieved from the Gene Expression Omnibus [17]. Subsequently, ceRNA-based transcriptional signatures were revealed via the construction of a T1DM-related ceRNA regulatory network.

## Methods

### Data source and annotation

Expression and non-coding RNA profiles (GEO accession number: GSE55100) deposited by Yang et al. [16] were downloaded from the National Center for Biotechnology Information GEO (https://www.ncbi.nlm.nih.gov/geo/) [18], which consist of two subseries, GSE55098 and GSE55099. The GSE55098 contains expression data of peripheral blood mononuclear cells from 12 patients with T1DM and 10 normal control subjects, which were based on the GPL570 [HG-U133_Plus_2] Affymetrix Human Genome U133 Plus 2.0 Array platform. The GSE55099 contains microRNA expression data from peripheral blood mononuclear cells from the same 12 patients with T1DM and 10 normal control subjects, based on the GPL8786 [miRNA-1] Affymetrix Multispecies miRNA-1 Array platform. The miRNAs, lncRNAs, and mRNAs in the downloaded profiles were annotated via the Human Genome Organization Gene Nomenclature Committee (http://www.genenames.org/) [19], where over 40,000 approved gene symbols have been recorded, of which more than 19,000 are for protein-coding genes.

### Identification of differentially expressed RNAs

The differentially expressed RNAs (DERs) between T1DM and normal controls were screened with Limma (Linear Models for Microarray Data) package (Version 3.34.0; https://bioconductor.org/packages/release/bioc/html/limma.html) [20] of R3.4.1. The cut-off criteria were set as false discovery rate (FDR) less than 0.05 and |log_2_ fold change| greater than 0.5. Two-way hierarchical clustering analysis based on Euclidean distance was executed for all the identified DERs via the pheatmap (Version 1.0.8, https://cran.r-project.org/web/packages/pheatmap/index.html) of R3.4.1 [21–23]. Meanwhile, gene ontology (GO) functional enrichment in terms of biological process as well as Kyoto Encyclopedia of Genes and Genomes (KEGG) pathway enrichment analysis were conducted for differentially expressed mRNAs via the Database for Annotation, Visualization and Integrated Discovery (DAVID, Version 6.8, https://david.ncifcrf.gov/) [24, 25], with a threshold of *p* < 0.05.

### Construction of ceRNA regulatory network

The regulatory interactions between differentially expressed lncRNAs and miRNAs were retrieved from the DIANA-LncBase (Version 2, http://carolina.imis.athena-innovation.gr/diana_tools/web/index.php) [17]. The negative regulatory interactions with a miRNA target gene score (miTG-score) larger than 0.6 (the default threshold in DIANA-LncBase) were retained to construct the lncRNA–miRNA regulatory network, which was visualized with Cytoscape (Version 3.6.1, https://cytoscape.org/) [26].

The target genes of differentially expressed miRNAs were obtained by using starBase (Version 2.0, http://starbase.sysu.edu.cn/), which contains prediction data from the five databases of targetScan, picTar, RNA22, PITA, and miRanda [25]. The negative regulatory interactions predicted in at least one of the five databases were retained to construct the miRNA–mRNA regulatory network, which was visualized with Cytoscape (Version 3.6.1, https://cytoscape.org/) [26].

Afterwards, the lncRNA–miRNA–mRNA (1) regulatory network was constructed by integrating the above lncRNA–miRNA regulatory interactions with the miRNA–mRNA regulatory interactions, and visualized with Cytoscape (Version 3.6.1, https://cytoscape.org/) [26]. Moreover, GO functional enrichment in terms of biological process and pathway enrichment analysis were conducted for genes in the ceRNA regulatory network via DAVID (Version 6.8, https://david.ncifcrf.gov/) [24, 25], and *p* < 0.05 was used as the cut-off criterion.

### T1DM-related ceRNA regulatory network

The miRNAs associated with T1DM were obtained by using the Human microRNA Disease Database (HMDD; Version 3.2, http://www.cuilab.cn/hmdd) [27], with the option “Disease name” filled with “type 1 diabetes mellitus.” The disease-related miRNAs were mapped onto the ceRNA regulatory network to reveal the target genes for pathway enrichment analysis via DAVID (Version 6.8, https://david.ncifcrf.gov/) [24, 25] and *p* < 0.05 was used as the cut-off criterion. In addition, the sequences of interested lncRNAs and miRNAs were downloaded from Ensembl genome browser (http://asia.ensembl.org/index.html) and miRBase (http://www.mirbase.org/), respectively. Binding sites between lncRNAs and miRNAs were predicted by using miRanda (http://cbio.mskcc.org/miRNA2003/miranda.html) [28], which is an algorithm for finding genomic targets for microRNAs.

On the other hand, the KEGG pathways associated with T1DM were screened from the Comparative Toxicogenomics Database update 2019 (http://ctd.mdibl.org/) [29], with the keyword “type 1 diabetes mellitus.” The enriched KEGG pathways from DAVID were compared with those obtained from the Comparative Toxicogenomics Database (CTD).

## Results

### Data annotation and DER screening

**Fig. 1:**
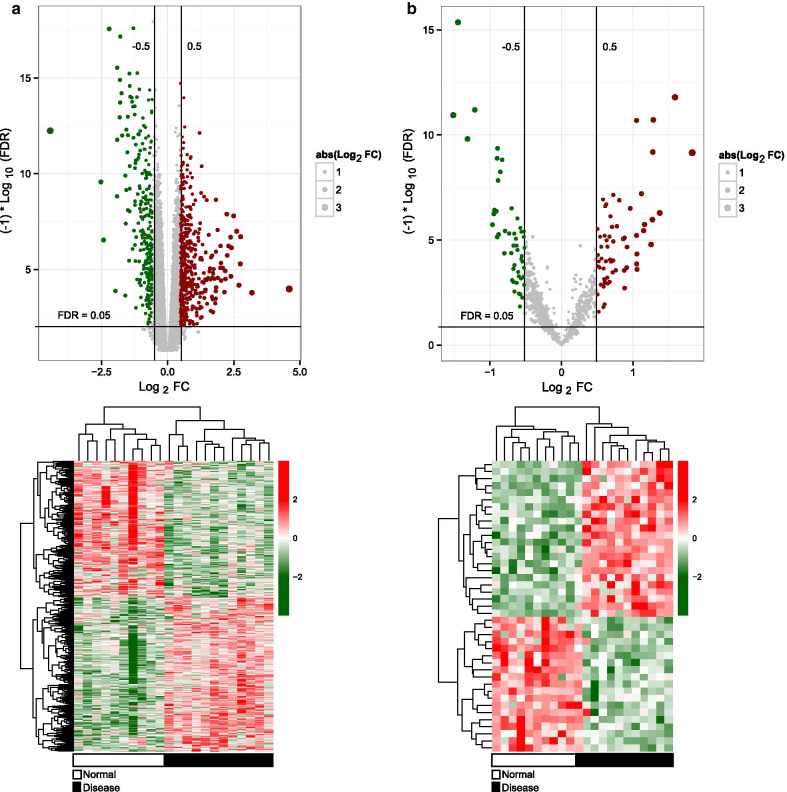
Identification and hierarchical clustering analysis of differentially expressed RNAs (DERs) in peripheral blood mononuclear cell samples between 12 type 1 diabetes mellitus (T1DM) patients and 10 normal control individuals. **a** Identified differentially expressed lncRNAs and mRNAs with thresholds of FDR less than 0.05 and |log_2_ fold change (FC)| greater than 0.5. Blue dots represent downregulated DERs and red dots represent upregulated DERs. **b** Identified differentially expressed miRNAs with thresholds of FDR < 0.05 and |log_2_FC|> 0.5. Blue dots represent downregulated DERs and red dots represent upregulated DERs

According to the platform annotation information, 946 lncRNAs, 597 miRNAs, and 10,085 mRNAs were received. By comparing the sample profiles of patients with T1DM with those of normal controls, 926 DERs were identified using Limma package, including 847 mRNAs (448 downregulated and 399 upregulated), 41 lncRNAs (22 downregulated and 19 upregulated), and 38 miRNAs (18 downregulated and 20 upregulated; Fig. 1). Moreover, the hierarchical clustering analysis revealed that all mRNAs and lncRNAs identified could adequately distinguish between T1DM samples and normal samples (Fig. 1a). Meanwhile, T1DM samples could also be discriminated from normal samples based on the expression levels of the 38 differentially expressed miRNAs (Fig. 1b).

**Fig. 2:**
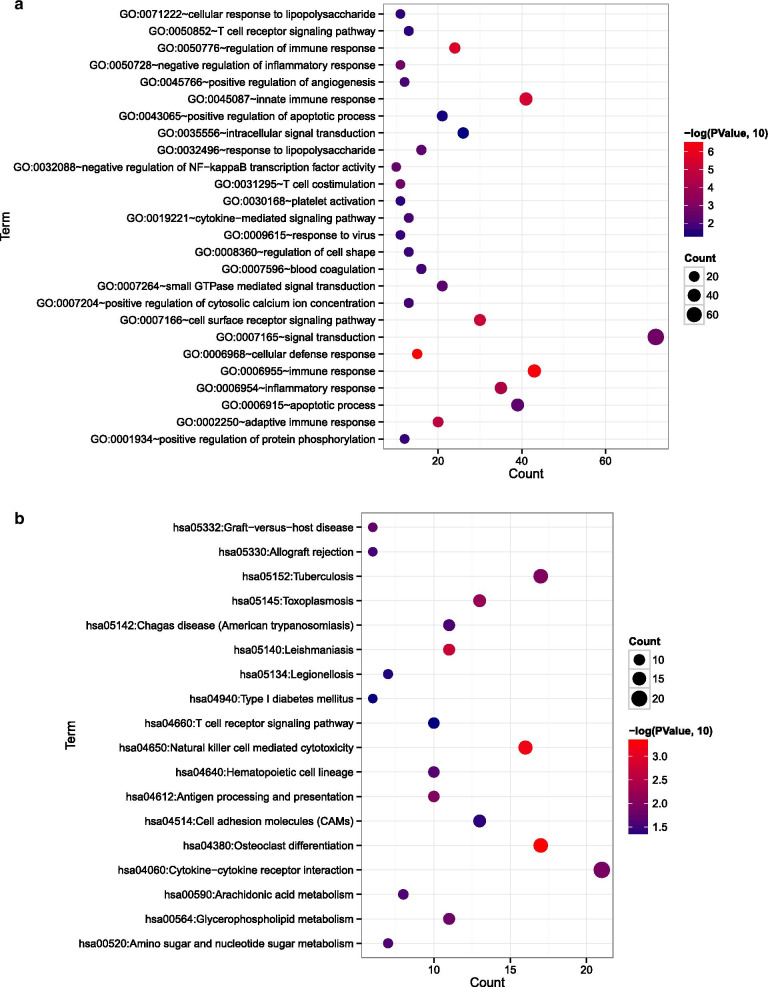
Gene ontology (GO) functional enrichment in terms of biological process and Kyoto Encyclopedia of Genes and Genomes (KEGG) pathway enrichment analysis for differentially expressed mRNAs. **a** Enriched GO terms for differentially expressed mRNAs. **b** Enriched KEGG pathways for differentially expressed mRNAs

The GO functional enrichment analysis indicated that the above differentially expressed mRNAs were significantly associated with the biological processes of cellular defense response (*p* = 3.020E−07), immune response (*p* = 4.320E−07), regulation of immune response (*p* = 2.730E−06), innate immune response (*p* = 4.610E-06), cell surface receptor signaling pathway (*p* = 8.640E−06), adaptive immune response (*p* = 2.170E−05), and inflammatory response (*p* = 5.040E−05; Additional file 1: Table S1, Fig. 2a). At the same time, differentially expressed mRNAs were enriched in 18 KEGG pathways, such as osteoclast differentiation (*p* = 4.930E−04), natural-killer-cell-mediated cytotoxicity (*p* = 6.850E−04), antigen processing and presentation (*p* = 1.008E−02), cytokine–cytokine receptor interaction (*p* = 1.250E−02), and glycerophospholipid metabolism (*p* = 1.520E−02; Additional file 1: Table S1, Fig. 2b).

### Constructed ceRNA regulatory network

The regulatory interactions between differentially expressed lncRNAs and differentially expressed miRNAs were acquired by using the DIANA-LncBase version 2 database. The 94 pairs of negative lncRNA–miRNA regulatory interactions with a coefficient larger than 0.6 were retained to build the lncRNA–miRNA regulatory network, which consisted of 61 nodes, 36 miRNAs, and 25 lncRNAs (Additional file 2: Figure S1). The target genes of differentially expressed miRNAs were predicted using starBase. Afterwards, the 915 pairs of negative miRNA–mRNA regulatory interactions involving differentially expressed mRNAs were submitted to construct the miRNA–mRNA regulatory network, which consisted of 322 nodes, 23 miRNAs, and 299 mRNAs (Additional file 3: Figure S2).

**Fig. 3:**
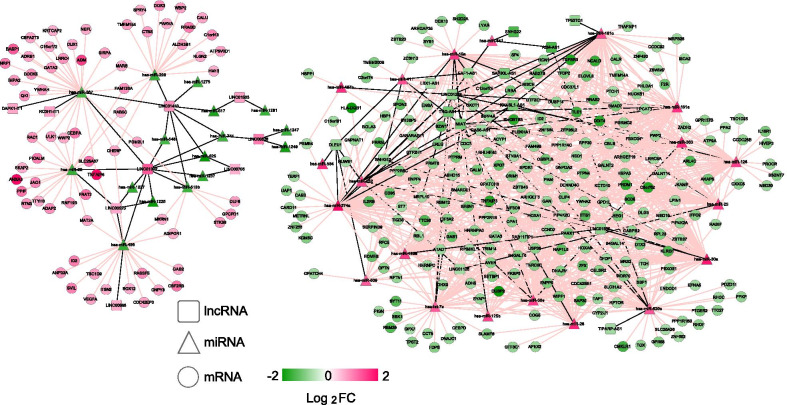
The constructed competing endogenous RNA (1) regulatory network. Square, triangle, and circle nodes represent lncRNAs, miRNAs, and mRNAs, respectively. Nodecolors range from green to red, which indicate downregulated to upregulated expression changes

**Fig. 4:**
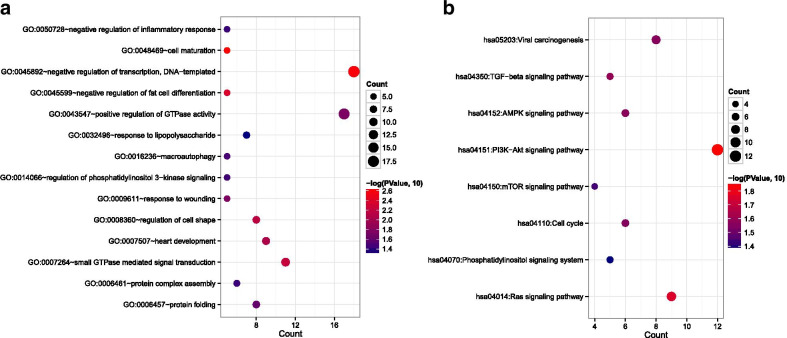
GO functional enrichment in terms of biological process and KEGG pathway enrichment analysis for mRNAs in the ceRNA regulatory network. **a** Enriched GO terms for mRNAs in the ceRNA regulatory network. **b** Enriched KEGG pathways for mRNAs in the ceRNA regulatory network

By integrating the lncRNA–miRNA regulatory interactions with miRNA–mRNA regulatory interactions, the lncRNA–miRNA–mRNA (1) regulatory network was established (Fig. 3). There were 360 nodes and 1,002 regulatory interactions in the ceRNA regulatory network, including 12 lncRNAs, 10 miRNAs, and 24 mRNAs. The mRNAs in the ceRNA regulatory network were substantially related to 14 GO biological processes, such as cell maturation (*p* = 2.434E−03), negative regulation of transcription, DNA templated (*p* = 2.598E−03), negative regulation of fat cell differentiation (*p* = 4.295E−03), and small GTPase–mediated signal transduction (*p* = 5.980E−03; Fig. 4a, Additional file 4: Table S2). Meanwhile, eight KEGG pathways were enriched for the mRNAs in the ceRNA regulatory network, including the PI3K-Akt signaling pathway (*p* = 1.442E−02), the Ras signaling pathway (*p* = 1.753E−02), and the TGF-beta signaling pathway (*p* = 2.579E−02; Fig. 4b, Additional file 4: Table S2).

### T1DM-related ceRNA regulatory network

**Fig. 5:**
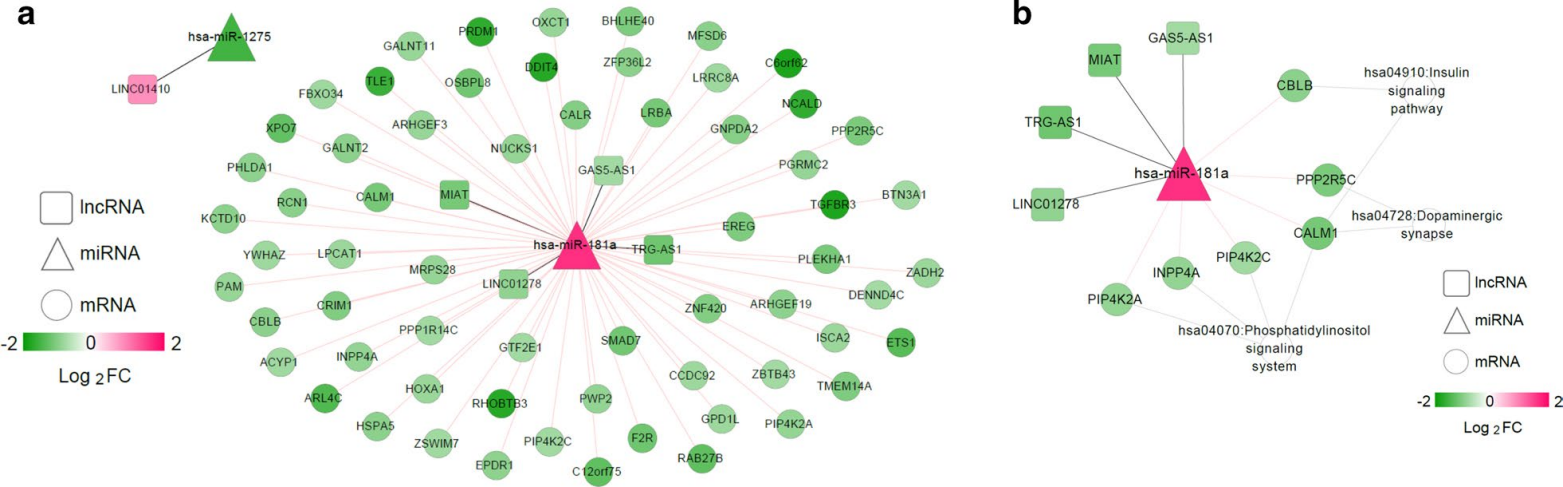
T1DM-related ceRNA regulatory network and disease pathway network. **a** The T1DM-related ceRNA regulatory network. **b** The disease pathway network. Square, triangle, and circle nodes represent lncRNAs, miRNAs, and mRNAs, respectively. Nodecolors range from green to red, which indicate downregulated to upregulated expression changes

The miRNAs related to T1DM were downloaded from HMDD Version 3.2 with the disease name “type 1 diabetes mellitus,” and 32 miRNAs were obtained. By comparing with the miRNAs in the ceRNA regulatory network, two overlapping miRNAs (*hsa-miR-181a* and *hsa-miR-1275*) were retained, and the regulatory network involving these two miRNAs was separated from the ceRNA regulatory network (Fig. 5a). The genes in the separated regulatory network were significantly enriched in seven pathways, including the phosphatidylinositol signaling system (*p* = 4.567E−04), inositol phosphate metabolism (*p* = 2.504E−03), mucin type O-Glycan biosynthesis (*p* = 1.029E−02), the ErbB signaling pathway (*p* = 2.636E−02), glycerophospholipid metabolism (*p* = 2.842E−02), dopaminergic synapse (*p* = 3.634E−02), and the insulin signaling pathway (*p* = 3.856E−02; Table 1). Binding sites of *hsa-miR-181a* with *GAS5-AS1*, *LINC01278*, and *MIAT* were exhibited in Additional file 5: Figure S3. The possible binding sites of *hsa-miR-1275* with *LINC01410* were shown in the Additional file 6: Figure S4.

**Table 1.** Seven significantly enriched Kyoto Encyclopedia of Genes and Genomes (KEGG) pathways for mRNAs in type 1 diabetes mellitus (T1DM) related competing endogenous (ceRNA) regulatory network

Term	Count	*P* value	Genes
*hsa04070:Phosphatidylinositol signaling system	4	4.567E−04	*INPP4A*,* PIP4K2A*,* PIP4K2C*,* CALM1*
hsa00562:Inositol phosphate metabolism	3	2.504E−03	*INPP4A*,* PIP4K2A*,* PIP4K2C*
hsa00512:Mucin type O-Glycan biosynthesis	2	1.029E−02	*GALNT2*,* GALNT11*
hsa04012:ErbB signaling pathway	2	2.636E−02	*CBLB*,* EREG*
hsa00564:Glycerophospholipid metabolism	2	2.842E−02	*GPD1L*,* LPCAT1*
*hsa04728:Dopaminergic synapse	2	3.634E−02	*PPP2R5C*,* CALM1*
*hsa04910:Insulin signaling pathway	2	3.856E−02	*CBLB*,* CALM1*

*The pathway overlapping with T1DM-related pathways retrieved from Comparative Toxicogenomics Database (CTD)

The KEGG pathways related to T1DM were further searched in the CTD with the keyword “type 1 diabetes mellitus,” and 148 KEGG pathways were revealed. Three overlapping KEGG pathways were revealed by comparing these 148 KEGG pathways with the above seven enriched pathways, including the phosphatidylinositol signaling system, dopaminergic synapse, and the insulin signaling pathway. The disease pathway network showed that four downregulated lncRNAs (*LINC01278*, *TRG-AS1*, *MIAT*, and *GAS5-AS1*) could regulate overexpressed *hsa-miR-181a*, which had six downregulated target genes that were involved in the three KEGG pathways (Fig. 5b). *PIP4K2A*, *INPP4A*, *PIP4K2C*, and *CALM1* were involved in the phosphatidylinositol signaling system. *CBLB* and *CALM1* were related to the insulin signaling pathway. In the dopaminergic synapse, *CALM1* and *PPP2R5C* were enriched.

## Discussion

Selective destruction of insulin-producing pancreatic islet β cells causes T1DM, a chronic immune-mediated and inflammatory disease [30]. In recent years, increasing efforts have been made to understand the molecular mechanisms of the pathogenesis of T1DM; and, researching ceRNA regulatory network-based RNA signatures may contribute to gaining further insights into its specific regulatory roles in T1DM. In our study, 926 DERs were identified in expression profiles of peripheral blood mononuclear cells from T1DM patients, including 847 mRNAs, 41 lncRNAs, and 38 miRNAs. In the study of Yang et al. [16] 24 miRNAs and 1218 genes were found differently expressed in patients with newly diagnosed T1DM. Expression levels of *hsa-let-7a*, *hsa-miR-1275*, *hsa-miR-22*, *hsa-miR-25*, *hsa-miR-28*, and *hsa-miR-486*, which were identified as differentially expressed miRNAs by Yang et al., were also found significantly different in our study. Liu et al. [31] have downloaded GSE55100 and differentially expressed miRNA-mRNA interactions were unveiled from the 7 miRNAs (*hsa-miR-374a*, *hsa-miR-146b*, *hsa-miR-181a*, *hsa-miR-19b*, *hsa-miR-125b*, *hsa-let-7f*, and *hsa-miR28*) and 651 mRNAs. In our study, differentially expressed miRNA-mRNA regulatory interactions involved 23 miRNAs and 299 mRNAs, and 4 miRNAs (*hsa-miR-374a*, *hsa-miR-181a*, *hsa-miR-125b*, and *hsa-miR28*) were consistent with the previous study. In order to revel potential hub genes involved in the pathogenesis of Chinese type 1 diabetic patients, GSE55100 has been also downloaded to identify DEGs, and thirteen hub genes (*MMP9*, *ARG1*, *CAMP*, *CHI3L1*, *CRISP3*, *SLPI*, *LCN2*, *PGLYRP1*, *LTF*, *RETN*, *CEACAM1*, *CEACAM8*, and *MS4A3*) were retrieved by module analysis [32]. These thirteen hub genes were also identified as differentially expressed genes in our study.

Besides, two miRNAs (*hsa-miR-181a* and *hsa-miR-1275*) were screened as T1DM-related miRNAs based on information from the HMDD to construct the T1DM-related ceRNA regulatory network. The genes in the T1DM-related ceRNA regulatory network were significantly enriched in seven pathways, and three overlapping pathways, including the phosphatidylinositol signaling system, dopaminergic synapse, and the insulin signaling pathway,were revealed by comparing with T1DM-related pathways in the CTD. The disease pathway network contained one upregulated miRNA (*hsa-miR-181a*) and four lncRNAs (*LINC01278*, *TRG-AS1*, *MIAT*, and *GAS5-AS1*). Overexpression of *miR-181a* levels were found in insulin-resistant cultured hepatocytes, and inhibition of *miR-181a* may lead to increased protein levels of SIRT1, which is a potential therapeutic target for combating insulin resistance, and thereby improving hepatic insulin sensitivity [33]. It is well known that hyperglycaemia, acidosis, and insulin resistance play significant roles in T1DM [34]. In a study by Nielsen et al., 12 upregulated miRNAs, including *miR-152*, *miR-30a-5p*, *miR-181a*, *miR-24*, *miR-148a*, *miR-210*, *miR-27a*, *miR-29a*, *miR-26a*, *miR-27b*, *miR-25*, and *miR-200a*, were identified in T1DM patients [35]. Thus, the changes in expression of *hsa-miR-181a* in this study were consistent with the results of previous studies.

Moreover, six target genes (*PIP4K2A*, *INPP4A*, *PIP4K2C*, *CALM1*, *CBLB*, and *PPP2R5C*) of *hsa-miR-181a* were involved in a further established disease pathway network based on the T1DM-related ceRNA regulatory network. T1DM arises from autoimmune-mediated β-cell destruction, and this defect is closely associated with the molecular levels found in the insulin signaling pathway [36, 37]. Casitas B-lineage lymphoma b (*CBLB*), a member of the CBL/SLI family of ubiquitin-protein ligases, functions as a key regulator of lymphocyte activation and autoimmunity [38, 39]. Komeda diabetes-prone rats, which are a spontaneous animal model of human type 1 diabetes, as well as *CBLB*-deficient mice, have been observed to have infiltrations of lymphocytes into pancreatic islets, the thyroid gland, and kidneys, suggesting that *CBLB* dysfunction leads to autoimmune processes [40, 41]. Transgenic complementation with wild type *CBLB* greatly suppresses the development of the Komeda diabetes-prone phenotype, indicating that *CBLB* is a negative regulator of autoimmunity and a susceptibility gene for T1DM in the rat [42]. Moreover, one single nucleotide polymorphism in exon 12 of the *CBLB* gene has been shown to be associated with T1DM based on analysis of a large Danish T1DM database of 480 families [43]. In our study, expression levels of *CBLB* were also lower in the peripheral blood mononuclear cell profiles of T1DM patients. Thus, *CBLB* is a key gene of T1DM, and downregulated *CBLB* participating in the insulin signaling pathway may contribute to the autoimmune disease of T1DM. In addition, regulatory interactions between four lncRNAs (*LINC01278*, *TRG-AS1*, *MIAT*,and *GAS5-AS1*) and *hsa-miR-181a* were revealed in the T1DM-related ceRNA regulatory network. However, there have been no reports, to our knowledge, of the roles of *LINC01278*, *TRG-AS1*, *MIAT*, and *GAS5-AS1* in the pathogenesis of T1DM. According to the ceRNA hypothesis, it can be surmised that these four lncRNAs (*LINC01278*, *TRG-AS1*, *MIAT*, and *GAS5-AS1*) might participate in regulating the expression levels of target genes of *hsa-miR-181a* by competing with *hsa-miR-181a.*

## Conclusion

In conclusion, 847 mRNAs, 41 lncRNAs, and 38 miRNAs were significantly differentially expressed in peripheral blood mononuclear cells of T1DM patients. A disease pathway network was made based on the T1DM-related ceRNA regulatory network, which consisted of four lncRNAs (*LINC01278*, *TRG-AS1*, *MIAT*, and *GAS5-AS1*), one miRNA (*hsa-miR-181a*), six target genes, as well as three important pathways related to T1DM, including the phosphatidylinositol signaling system (involving *PIP4K2A*, *INPP4A*, *PIP4K2C*, and *CALM1*), the dopaminergic synapse (involving *CALM1* and *PPP2R5C*), and the insulin signaling pathway (involving *CBLB* and *CALM1*). These results suggest that the signature RNAs identified above may serve as important regulators in the pathogenesis of T1DM. However, the expression changes of miRNA and mRNAs should be further detected in different patient cohort by using laboratory experiments. More particularly, four lncRNAs, *LINC01278*, *TRG-AS1*, *MIAT*, and *GAS5-AS1*, may compete with *hsa-miR-181* to regulate its target genes in T1DM.

## Supplementary Information

**Additional file 1: Table S1.** The significantly enriched gene ontology (GO) biological processes and Kyoto Encyclopedia of Genes and Genomes (KEGG) pathways for differentially expressed mRNAs.

**Additional file 2: Figure S1.** The constructed lncRNA–miRNA regulatory network. Square and triangle nodes represent lncRNAs and miRNAs, respectively. Nodecolors range from green to red, which indicate downregulated to upregulated expression changes.

**Additional file 3: Figure S2.** The constructed miRNA–mRNA regulatory network. Triangle and circle nodes represent miRNAs and mRNAs, respectively. Nodecolors range from green to red, which indicate downregulated to upregulated expression changes. The phosphatidylinositol signaling system, dopaminergic synapse, and the insulin signaling pathway were the three major pathways related to T1DM.

**Additional file 4: Table S2.** The significantly enriched gene ontology (GO) biological processes and Kyoto Encyclopedia of Genes and Genomes (KEGG) pathways for mRNAs in the competing endogenous (1) regulatory network.

**Additional file 5: Figure S3.** Binding sites of *hsa-miR-181a* with *GAS5-AS1*, *LINC01278*, and *MIAT*.

**Additional file 6: Figure S4.** Possible binding sites of *hsa-miR-1275* with *LINC01410*.

## Data Availability

GSE55100 (https://www.ncbi.nlm.nih.gov/geo/query/acc.cgi?acc=GSE55100) contains two subseries GSE55098 (https://www.ncbi.nlm.nih.gov/geo/query/acc.cgi?acc=GSE55098)) based on the GPL570 ([HG-U133_Plus_2] Affymetrix Human Genome U133 Plus 2.0 Array) and GSE55099 (https://www.ncbi.nlm.nih.gov/geo/query/acc.cgi?acc=GSE55099) based on the GPL8786 ([miRNA-1] Affymetrix Multispecies miRNA-1 Array), were downloaded from the National Center for Biotechnology Information (NCBI) Gene Expression Omnibus (GEO, https://www.ncbi.nlm.nih.gov/geo/). The raw data were collected and analyzed by the Authors, and are not ready to share their data because the data have not been published.

## Abbreviations

T1DM: Type 1 diabetes mellitus
lncRNAs: Long non-coding RNAs
miRNAs: MicroRNAs
CTD: Comparative Toxicogenomics Database
ceRNAs: Competing endogenous RNAs
DERs: Differentially expressed RNAs
GO: Gene ontology
KEGG: Kyoto Encyclopedia of Genes and Genomes
