# Supplementary figures and images for: Signature RNAS and related regulatory roles in type 1 diabetes mellitus based on competing endogenous RNA regulatory network analysis
Source: BMC Med Genomics. 2021 May 18;14:133. doi: 10.1186/s12920-021-00931-0 (PMC8130321; doi:10.1186/s12920-021-00931-0)

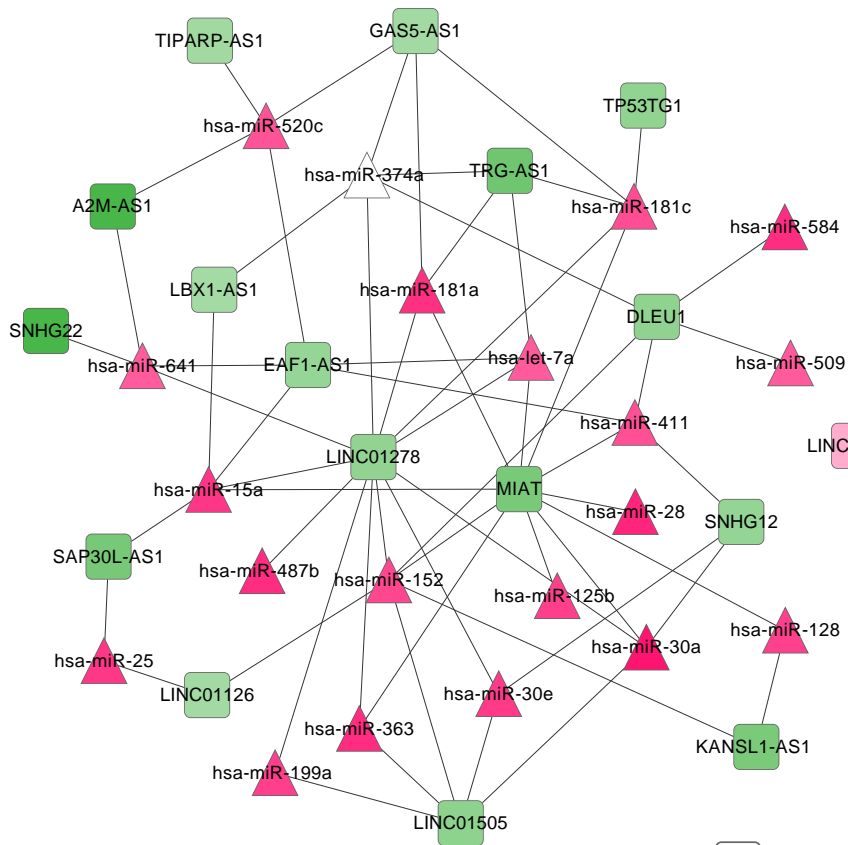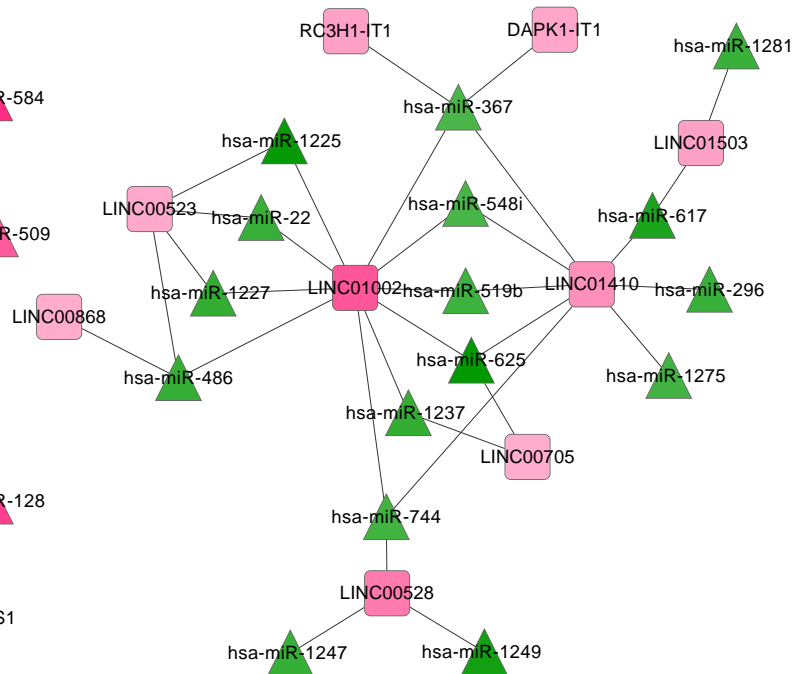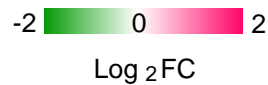

Supplement: Supplementary file 2 — Additional file 2: Figure S1. The constructed lncRNA–miRNA regulatory network. Square and triangle nodes represent lncRNAs and miRNAs, respectively. Nodecolors range from green to red, which indicate downregulated to upregulated expression changes. [file 12920_2021_931_MOESM2_ESM.pdf]

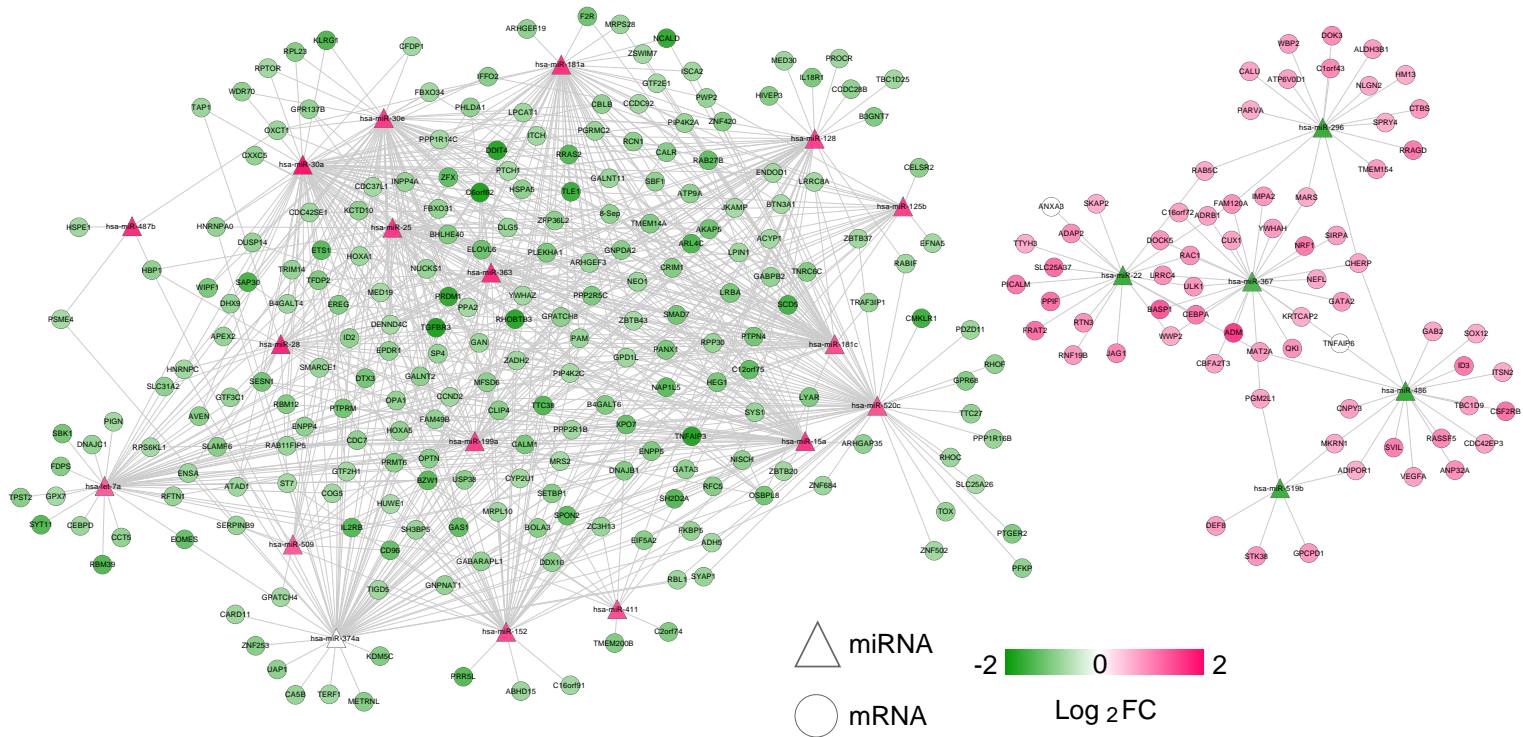

Supplement: Supplementary file 3 — Additional file 3: Figure S2. The constructed miRNA–mRNA regulatory network. Triangle and circle nodes represent miRNAs and mRNAs, respectively. Nodecolors range from green to red, which indicate downregulated to upregulated expression changes. The phosphatidylinositol signaling system, dopaminergic synapse, and the insulin signaling pathway were the three major pathways related to T1DM. [file 12920_2021_931_MOESM3_ESM.pdf]
