# Supplementary material for: Signature RNAS and related regulatory roles in type 1 diabetes mellitus based on competing endogenous RNA regulatory network analysis
Source: BMC Med Genomics. 2021 May 18;14:133. doi: 10.1186/s12920-021-00931-0 (PMC8130321; doi:10.1186/s12920-021-00931-0)
Supplement: Supplementary file 5 — Additional file 5: Figure S3. Binding sites of hsa-miR-181a with GAS5-AS1, LINC01278, and MIAT. [file 12920_2021_931_MOESM5_ESM.pdf]

Seq1	Seq2	Tot Score	Tot Energ	Seq1 Position	Seq2 Position	Length
hsa-miR-181a	GAS5-AS1	83	-22.52	3 22	589 607	19

```
Query:  3' UGAGUGGCUGUCGCAACUUACAA 5'
             || ||| | | ||||||||
Ref:    5' CGTC-CCGCCCGGGTTGGGTGGG 3'
```

Seq1	Seq2	Tot Score	Tot Energ	Seq1 Position	Seq2 Position	Length
hsa-miR-181a	LINC01278	81	-20.12	1 24	872 897	25

```
Query:  3' UGAGUGGCUGUCGCAA--CUUACAA 5'
           :|  :|||:  |||||  ||||| |
Ref:    5' GCGAGCCGGGCGCGTTCAGGGTGGT 3'
```

Seq1	Seq2	Tot Score	Tot Energ	Seq1 Position	Seq2 Position	Length
hsa-miR-181a	MIAT	73	-20.45	4 23	135 153	19

```
Query:  3' UGAGUGGCUGUCGCAACUUACAA 5'
              ||||| ||||  ||| |||
Ref:    5' TGACACCGTCAGC-ATGGCTGTA 3'
```
