# Supplementary material for: Signature RNAS and related regulatory roles in type 1 diabetes mellitus based on competing endogenous RNA regulatory network analysis
Source: BMC Med Genomics. 2021 May 18;14:133. doi: 10.1186/s12920-021-00931-0 (PMC8130321; doi:10.1186/s12920-021-00931-0)
Supplement: Supplementary file 6 — Additional file 6: Figure S4. Possible binding sites of hsa-miR-1275 with LINC01410. [file 12920_2021_931_MOESM6_ESM.pdf]

Seq1	Seq2	Tot Score	Tot Energy	Seq1 Position	Seq2 Position	Length
hsa-miR-1275	LINC01410	100	-28.63	1 18	1197 1218	21

```
Query:     3’ CUGUC---GGAGAGG-GGGUG 5’
              |:|:|   ||||||| |||||
Ref:       5’ GGCGGCCTCCTTTCCGCCCAC 3’
```

Seq1	Seq2	Tot Score	Tot Energy	Seq1 Position	Seq2 Position	Length
hsa-miR-1275	LINC01410	94	-22.91	1 18	1159 1178	19

```
Query:     3’ CUGUCGGAG--AGGGGGUG 5’
              |: :|||||  ||||||||
Ref:       5’ GGAGGCCTCTGTCCCCTGC 3’
```

Seq1	Seq2	Tot Score	Tot Energy	Seq1 Position	Seq2 Position	Length
hsa-miR-1275	LINC01410	93	-26.16	1 18	1037 1056	19

```
Query:     3’ CUGUCGG-AGAGGG-GGUG 5’
              |:||||| |||||| ||||
Ref:       5’ GGCAGCTGTCTCCCTCTAC 3’
```

Seq1	Seq2	Tot Score	Tot Energy	Seq1 Position	Seq2 Position	Length
hsa-miR-1275	LINC01410	92	-20.04	2 17	1704 1721	17

```
Query:     3’ CUGUCG--GAGAGGGGGUG 5’
               |: ||  ||||||||||
Ref:       5’ TATTGCTTCTCTCTCCCGG 3’
```

Seq1	Seq2	Tot Score	Tot Energy	Seq1 Position	Seq2 Position	Length
hsa-miR-1275	LINC01410	92	-20.04	2 17	1704 1721	17

```
Query:     3’ CUGUCGGA--GAGGGGGUG 5’
              |:| ||||  || |||||
Ref:       5’ GGC-GCCTGGCTGCCCCAA 3’
```
